# The Role of Climate Change and Its Sensitivity on Long-Term Standardized Precipitation Evapotranspiration Index, Vegetation and Drought Changing Trends over East Asia

**DOI:** 10.3390/plants13030399

**Published:** 2024-01-29

**Authors:** Shahzad Ali, Abdul Basit, Muhammad Umair, Tyan Alice Makanda, Mohammed Rafi Shaik, Mohammad Ibrahim, Jian Ni

**Affiliations:** 1 College of Life Sciences, Zhejiang Normal University, Jinhua 321004, China; 2 College of Chemistry and Materials Science, Zhejiang Normal University, Jinhua 321004, China; 3 Department of Agriculture, Hazara University, Mansehra 21120, Pakistan; 4 School of Computer Science and Technology, Qingdao University, Qingdao 266109, China; 5 Department of Chemistry, College of Science, King Saud University, Riyadh 11451, Saudi Arabia; mrshaik@ksu.edu.sa; 6 Department of Chemistry, Abdul Wali Khan University, Mardan 23200, Pakistan; dribrahim@awkum.edu.pk

**Keywords:** drought analysis, seasonal SPEI, dry and wet conditions, vegetation dynamics, SPEI annual trends, climate variation, East Asia

## Abstract

Droughts have become more severe and frequent due to global warming. In this context, it is widely accepted that for drought assessments, both water supply (rainfall) and demand (standardized precipitation evapotranspiration index, SPEI) should be considered. Using SPEI, we explored the spatial-temporal patterns of dry and wet annual and seasonal changes in five sub-regions of East Asia during 1902–2018. These factors are linked to excess drought frequency and severity on the regional scale, and their effect on vegetation remains an important topic for climate change studies. Our results show that the SPEI significantly improved extreme drought and mostly affected the SPEI-06 and SPEI-12 growing seasons in East Asia during 1981–2018. The dry and wet annual SPEI trends mostly affect the five sub-regions of East Asia. The annual SPEI had two extremely dry spells during 1936–1947 and 1978–2018. Japan, South Korea, and North Korea are wet in the summer compared to other regions of East Asia, with drought frequency occurring at 51.4%, respectively. The mean drought frequencies in China and Mongolia are 57.4% and 54.6%. China and Mongolia are the driest regions in East Asia due to high drought frequency and duration. The spatial seasonal analysis of solar radiation (SR), water vapor pressure (WVP), wind speed (WS), vegetation condition index (VCI), temperature condition index (TCI), and vegetation health index (VHI) have confirmed that the East Asia region suffered from maximum drought events. The seasonal variation of SPEI shows no clear drying trends during summer and autumn seasons. During the winter and spring seasons, there was a dry trend in East Asia region. During 1902–1990, a seasonal SPEI presented diverse characteristics, with clear wet trends in Japan, Mongolia, and North Korea in four different growing seasons, with dry trends in China and South Korea. During 1991–2018, seasonal SPEI presented clear dry trends in Japan, Mongolia, and North Korea in different growing seasons, while China and South Korea showed a wet trend during the spring, autumn, and winter seasons. This ecological and climatic mechanism provides a good basis for the assessment of vegetation and drought-change variations within East Asia. An understandings of long-term vegetation trends and the effects of rainfall and SPEI on droughts of varying severity is essential for water resource management and climate change adaptation. Based on the results, water resources will increase under global warming, which may alleviate the water scarcity issue in the East Asia region.

## 1. Introduction

In recent decades, droughts have become more severe and frequent in many regions around the world [1], mainly in the East Asia region [2]. Recently, East Asia (EA) has faced severe drought due to climate change, which is affecting water resources and agricultural production [3]. According to the Intergovernmental Panel on Climate Change (IPCC), the sixth assessment report states that EA is a hotspot where a warm climate creates a risk of extreme dry climate [4]. It is often very difficult to identify, predict, and mitigate drought due to high spatiotemporal variability and unstructured drought influence [5]. Jones and Moberg [6] report temperature increases (0.5–2.0 °C) over the past 150 years [7]. A simple definition of drought is a period in which water supplies do not meet water demand [8,9]. Thus, drought is unavoidable and detrimental to ecosystems and socioeconomic systems [10]. Regional evapotranspiration (ET) consists of complex constraints such as soil moisture, vegetation growth status, and meteorological conditions [11]. The ET directly affected the water supply capacity of surface ecosystems [12]. Moreover, as climate change and socioeconomic development have significantly altered regional water supply and demand [13], understanding the impact of changes in water supply and demand on drought dynamics and drought occurrence is important for drought management [14,15].

In general, the East Asian meteorological drought is mainly determined by the failure of the East Asia summer monsoon rainfall, which provided 78% of the total yearly precipitation, which is essential for billions of people in the regions [16]. Soil moisture storage, evapotranspiration rates, and crop yields can be linked to agricultural drought [17]. Drought risk in East Asia is changing with the recent observed and projected climate [18]. According to a report by UNICEF [19], groundwater levels have significantly decreased as a result of this drought. Drought identifications established on a particular indicator for drought assessment [20], and water demand-supply signs are important for studying global warming [21]. From historical norms, numerous indicators have been developed to recognize drought-related variables [22]. Among them, the SPEI is often used as a proxy indicator for meteorological drought. The SPEI is usually used because it describes the collective impact of water supply (rainfall) and demands potential evapotranspiration (PET) rather than rainfall alone [23].

Previous findings have demonstrated that land use and climate factors such as temperature, rainfall, solar radiation, wind speed, etc. [24] affect water energy availability and lead to spatio-temporal variations in the evapotranspiration (ET) processes [25]. Prolonged drought and the resulting shortages of water, food, feed, and energy lead to mass migration [26]. The drought index is known to characterize drought duration, frequency, scale/depth, and spatial extent [27,28]. Some of the standard drought indices in earlier research were the vegetation condition index (VCI), vegetation health index (VHI), temperature condition index (TCI), and SPEI [29]. The SPEI was developed to incorporate the effects of climate change on drought dynamics [30]. SPEI exploits the variance between PET and rainfall. SPEI also has the potential to recognize drought characteristics within the context of climate change, global warming, and potential evapotranspiration (PET) [31]. The SPEI has been shown to be more sensitive in capturing drought characteristics than the standardized precipitation index (SPI) and the scout drought index for arid regions [32]. Earlier studies have investigated SPEI at various timescales to understand drought dynamics under a global warming condition [33,34].

Numerous studies report the effect of climate change on the East Asia (EA) region, providing a full understanding of potential drought risks [35]. Given the sensitivity of drought to temperature and its variability on multiple timescales [36], SPEI has been widely used for global and regional-scale drought monitoring and forecasting [37,38]. Lee et al. [39] assessed the impact of climate change on drought characteristics by using SPI and SPEI time series for North Korea from 1981 to 2100 and found that vegetation plays an important role in annual and seasonal variability in maintaining a balanced ecosystem. Insufficient rainfall can cause water stress, while high temperatures can cause heat stress, which affects the vegetation health index (VHI) [40]. The vegetation index can determine the growth condition of vegetation in an ecosystem [41]. Gupta et al. [42] reported that a high vapor pressure deficit and low soil moisture improved the negative effect on plant health. Several research works have reported seasonal variations, spatial-temporal trends, and the study of drought by using the SPEI and the new composite drought index [43]. However, an inclusive analysis of drought evolution and the relative impact of rainfall on drought severity from various perspectives are required for further study [44].

A comprehensive drought assessment is essential to formulating effective drought policies and mitigation strategies. A detailed study of drought characteristics is important for policymakers when considering the chain of environmental and socioeconomic consequences. Here, we investigated changes in long-term drought trends from 1902 to 2018 and possible associations with vegetation and climate dynamics drivers across East Asia and its sub-regions. This study also investigates the impact of SPEI, vapor pressure deficit, and vegetation estimation approaches on the changing characteristics of extreme drought (dry/wet) under various timescales. Understanding the spatio-temporal variation of drought characteristics, such as duration, intensity, and frequency, in East Asia and analyzing the long-term 1901–2018 drought trend at different timescales.

## 2. Results and Discussion

### 2.1. Drought Analysis of SPEI

**Figure 1:**
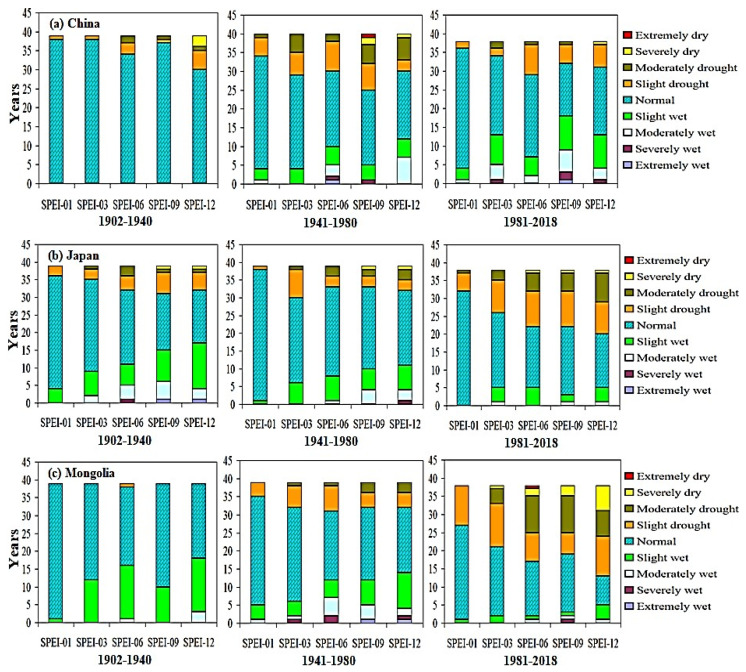
Drought analysis of SPEI for five different timescales across various countries of East Asia region during 1902–1940, 1941–1980, and 1981–2018.

**Figure 2:**
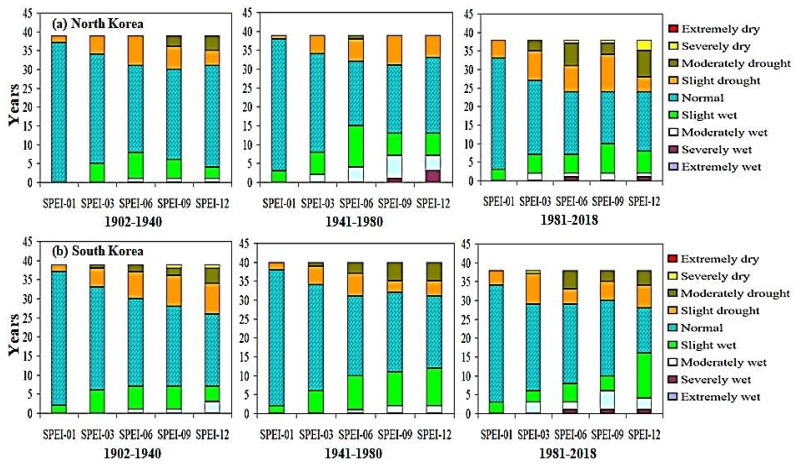
Drought analysis of SPEI for five different timescales across various countries of East Asia region during 1902–1940, 1941–1980, and 1981–2018.

The results suggest a consistent pattern of increasing frequency of dry events within the East Asian region from 1981 to 2018 (Figure 1 and Figure 2). Specifically, more drought events occurred during the 1981–2018 SPEI-06, SPEI-09, and SPEI-12 growing seasons compared to the 1902–1940 and 1941–1980 periods. All subregions of East Asia appear to have experienced increased levels of extreme aridity during various growing seasons from 1981 to 2018, but the most affected regions are Mongolia, South Korea, Japan, China, and North Korea. Drought is most severe in spring and summer, when temperatures increase and precipitation decreases [45], consistent with the findings of this paper. Related studies [46,47] show that the climate has warmed over the past 55 years and that precipitation has varied greatly. Declining rainfall and rising temperatures are the main reasons for the increase in droughts. For example, based on 6, 9, and 12-month SPEI values, the number of severe and extreme drought events in these regions nearly doubled between 1981 and 2018 compared to 1902–1940 and 1941–1980. Results showed that drought was most sensitive to longer SPEI timescales, suggesting that drought parameters have a greater influence on longer timescales than on shorter ones [48,49]. Improving in wet and dry events over the period 1981–2018 was significantly associated to a decrease as the percentage of rainy conditions. These anomalies appear to have increased in Mongolia, Japan, China, and Korea during the 1981–2018 SPEI-06, SPEI-09, and SPEI-12 growing seasons compared to 1902–1940. The cumulative effects of climate change could explain the increased drought associated with the long-term SPEI downward trend across large parts throughout the country [50,51]. Moderately drought events occurred in SPEI-09 and SPEI-12 during 1941–1980 and 1981–2018 period respectively over different sub regions of East Asia. In general, a more extreme drought occurred in the northwestern region of East Asia, followed by the southwestern region of East Asia. Results showed that drought was mostly susceptible to longer SPEI timescales, suggesting that drought parameters have a greater impact on longer timescales than on shorter timescales [52,53].

### 2.2. Temporal Annual Average SPEI Trends across East Asia

**Figure 3:**
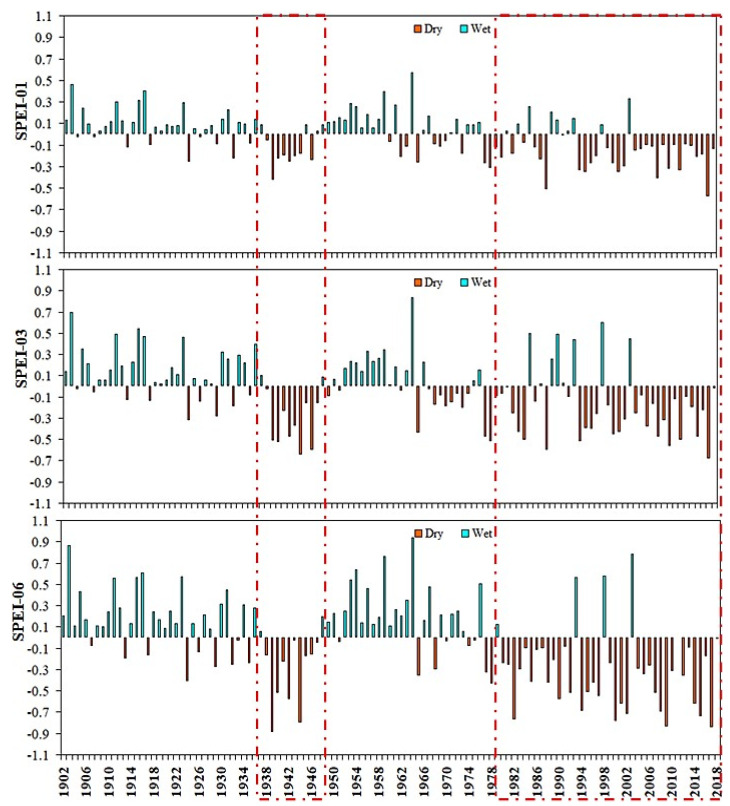
Annual average SPEI-01, SPEI-03, and SPEI-06 trends of the entire East Asia region from 1902 to 2018; red dashed frame mean have extreme dry years over East Asia.

**Figure 4:**
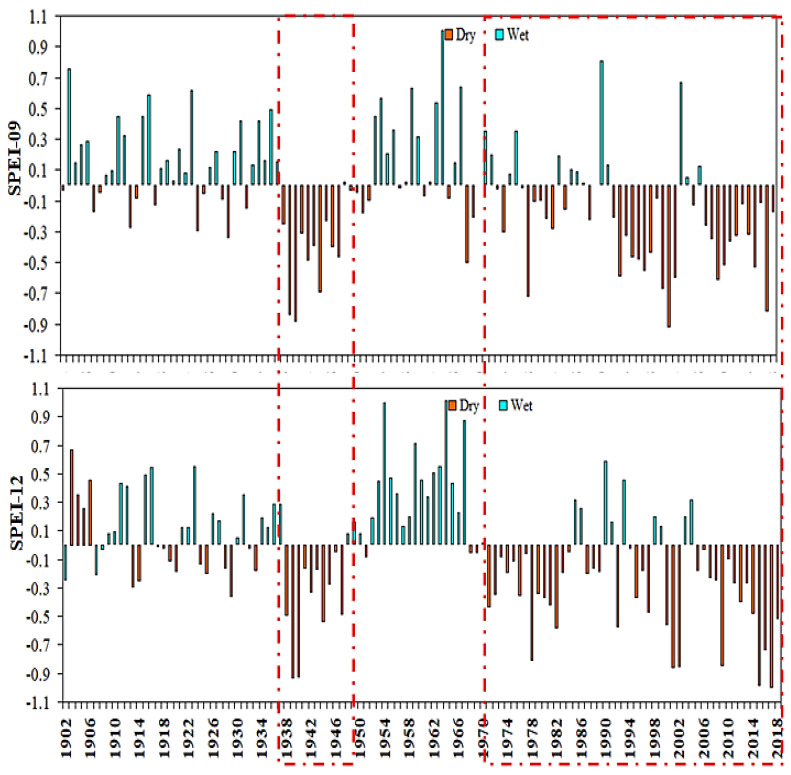
Annual average SPEI-09 and SPEI-12 trends of the entire East Asia region from 1902 to 2018; red dashed frame mean have extreme dry years over East Asia.

The annual time changes of SPEI during SPEI-01, SPEI-03, SPEI-06, SPEI-09, and SPEI-12 from 1902 to 2018 are shown in Figure 3 and Figure 4, respectively. The frequency of droughts has improved significantly over time, particularly since 1979. Drought events in East Asia have regional implications based on long-term SPEI values. On different timescales, the annual SPEI value changes significantly over time, and the change is slightly larger after 1979, which is more sensitive to land use and land cover changes. It is worth noting that the most extreme drought event (SPEI < −1.1, −ve) values indicate that drought stress was observed in all different growing seasons in East Asia after 1979. A potential reason for the increased drought trend in East Asia may be to reduce the rainfall trends [54,55].

A previous study found that the drought in 1966 was one of the worst droughts East Asia suffered, and the affected area under this drought was the highest [56,57]. Accordingly, the study also revealed that the highest spatial extent of drought, as evidence of drought stress was shown to exceed the maximum during SPEI-01, SPEI-03, SPEI-06, SPEI-09, and SPEI-12 (Figure 3 and Figure 4). The research results of drought change and vegetation response based upon the SPEI index are basically consistent with [58,59]. In addition, there were continuous drought years of 1938–1948 and 1979–2018. Similarly, when other timescales of SPEI are considered, such as 09 and 12 months, the number of extreme drought events was higher during 1970–2018. The temporal variation of SPEI-06 in the summer growing season of 1902–2018 (Figure 3) shows that East Asia experienced more drought periods. Cumulative impacts of climate change could explain increased drought associated with long-term SPEI downward trends over vast regions throughout the country [60,61]. Extreme drought events in East Asia based on September and December SPEI in 1938–1951, respectively, and 1968–2018 occurred twice. However, when considering the 09-month and 12-month SPEI, the number of extreme drought events was similar between the two study periods. It is worth mentioning that the region has recently experienced a decrease in precipitation and temperature warming trend [62,63]. For the rest of East Asia, the frequency of extreme droughts in SPEI-01 and SPEI-03 was generally lower than that in SPEI-06, SPEI-09, and SPEI-12 during 1902–2018, respectively.

### 2.3. Drought Frequency (DF)

Three phases (1902–1990, 1991–2018, and 1902–2018) were examined to effectively identify the drought evolution in various sub-regions of East Asia. By calculating the seasonal SPEI values of countries in East Asia, the spatial distribution of drought frequency in each season is shown in Table 1. Short-term drought is important for cultivated land, while long-term drought can affect the hydrological cycle [64,65]. From 1902 to 1990, the frequency of spring drought in Mongolia was 62.9%, mainly in China (59.0%); the frequency of summer drought in Mongolia was 64.3%, mainly in China (57.7%) and Japan (55.1%); the frequency of autumn drought in Mongolia occurred at 64.9%, mainly in Japan (56.4%); and the frequency of winter drought in Mongolia was 59.3%, mainly in China (51.8%). From 1991 to 2018, the frequency of spring drought in China was 47.6%, mainly in South Korea (47.0%); the frequency of summer drought in South Korea was 53.6%, the highest in China (56.5%); the frequency of autumn drought in China was 55.4%, mainly in South Korea (52.4%); and China’s winter drought frequency is 58.6%, mainly in Japan (48.2%). Wang et al. [37] divided the period 1961–2015 into three phases and found that both drought intensity and frequency increased from the first phase (1961–1996) to the second phase (1997–2002) and then decreased in the third phase (2003–2015). From 1902 to 2018, the frequency of spring drought in China was 56.3%, mainly in Mongolia (53.2%); the frequency of summer drought in China was 57.4%, mainly in Mongolia (54.6%); the frequency of autumn drought in China and Japan was 56.1%, mainly in Mongolia (53.5%); the frequency of winter drought was 53.4%, China, and Mongolia mainly occurred in Japan (50.1%). As mentioned above, the drought frequency in East Asia is higher in spring and summer, with an average of 52.4% and 53.3%, and lower in winter and autumn, with an average of 51.0% and 51.4%, respectively. Due to the rollback of water-saving measures and excessive deforestation in the early 1960s, soil erosion was severe, ecological and water circulation systems were damaged, and rainfall decreased [66,67,68].

### 2.4. Drought Duration (DD)

Shorter DD was associated with frequent but irregularly occurring short-term drought events, whereas longer DD indicated sustained and long-term drought events. Short-term droughts are important for agriculture, but long-term droughts affect the water cycle [69,70]. Table 2 shows the spatial distribution of DD in each season. During 1902–1990, the DD of the spring drought was 2.70 in Mongolia and mainly occurred in China (2.44); the DD of the summer drought was 2.80 in Mongolia, and the mainly lowest DD occurred in South Korea (2.05); the DD of the autumn drought was 2.85 in Mongolia and mainly occurred in China and Japan (2.29); the DD of the winter drought was 2.46 in Mongolia, while the significantly lowest DD occurred in Japan and South Korea (2.03). During 1991–2018, the DD of the spring drought was 1.91 in China and South Korea, and the lowest DD occurred in Mongolia (1.29); the maximum DD of the summer drought was 2.30 and 2.15 in China and South Korea, with the lowest value existing in Mongolia (1.29); the DD of the drought was 2.24 and 2.10 in China and South Korea, and the lowest DD occurred in Mongolia (1.21); the maximum DD of the winter drought was 2.42 in China, while the lowest DD occurred in Mongolia (1.43). Yadeta et al. [61] found that drought sequences identified from shorter timescales were of short duration and high frequency, whereas drought sequences identified from longer timescales were of lower frequency and longer duration. During 1902–2018, the highest DD of the spring drought was 2.29 in China, and the lowest occurred in North Korea (2.01); the DD of the summer drought was 2.35 in China and mainly occurred in Japan and North Korea (2.05); the DD of the autumn drought was 2.28 in China, while the lowest DD occurred in Japan and North Korea (1.98); and the DD of the winter drought was 2.10 in China and mainly occurred in North Korea (1.99). The spatial distribution shows that China and Mongolia each have higher values of DD in all four growing seasons. Moreover, the increasing trend of PET may be one of the signs of the recent drought [71,72,73].

### 2.5. Drought Intensity (DI)

Different study periods have different characteristics of spatial distribution. Table 3 shows the drought intensity for different study periods in East Asia. From 1902 to 1990, the DI of the spring drought in North Korea and South Korea was 0.48, while the DI in Mongolia was the lowest (0.37); the summer drought index in South Korea was 0.49, mainly in North Korea (0.48); the maximum DI of the autumn drought in South Korea was 0.50, while Mongolia The lowest DI (0.35). From 1991 to 2018, the highest spring drought DI was 0.78 in Mongolia and the lowest in China (0.52); the highest summer drought index in Mongolia was 0.76 and the lowest in China (0.43); the autumn drought index in China was 0.45, mainly in Mongolia and Japan (0.83); the lowest winter drought index in China is 0.41, and it mainly occurs in Mongolia (0.70). From 1902 to 2018, the DI of the spring drought in China was 0.44, which was significantly higher than 0.50 in Japan and South Korea; the summer drought index of Japan and North Korea was 0.49, the lowest in China (0.43); the DI of the autumn drought in China was 0.44, mainly in North Korea (0.51); and the winter drought DI is 0.47, while Japan, South Korea, and North Korea (0.50) have significant maximum values. From the spatial distribution, it can be seen that DI values are higher in Japan and South Korea in spring, Japan and South Korea in summer, South Korea in autumn, and Japan, South Korea, and North Korea in winter. Parts of the world are likely to experience an improvement in the frequency and intensity of daily temperatures and a decrease in extremely cold temperatures, leading to an increase in the length and intensity of warm periods [74,75,76].

### 2.6. Seasonal Average SR, WVP, WS, VCI, TCI, and VHI Distribution Values

**Figure 5:**
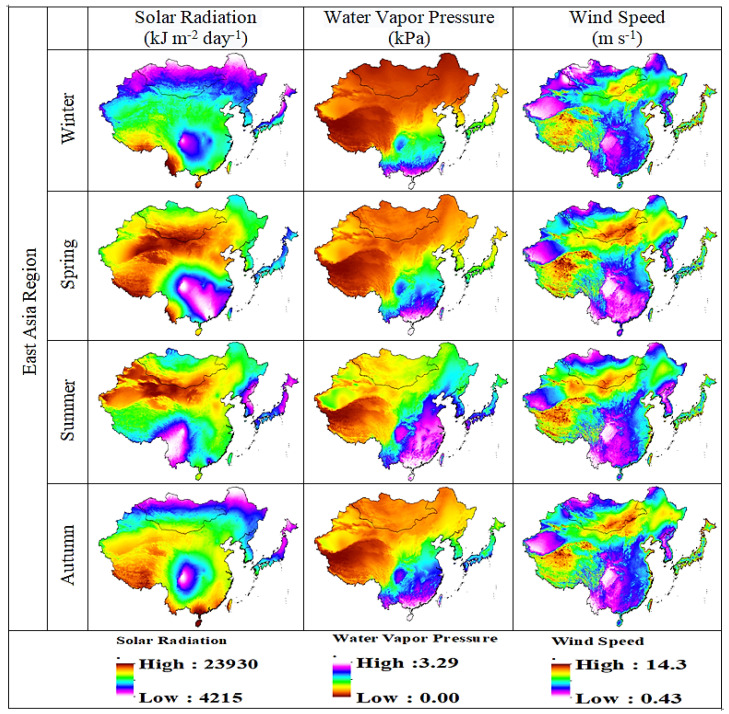
Spatial distribution of seasonal average SR, WVP, and WS values over East Asia.

In order to select the most suitable drought indicator for East Asia, the performance of three commonly used indicators, such as SR, WVP, and WS, was evaluated (Figure 5). In addition, the seasonal averages of SR, WVP, and WS were sequentially selected to verify the ability of drought monitoring. In addition, radiation can affect vegetation growth [77,78]. In different growing seasons, the spatial distribution analysis of the three different indicators was quite different. SR, WVP, and WS identified Northwest China and its surrounding areas as arid areas with high solar radiation, high wind speed, and low water vapor pressure. The temperature was responsible for 64% of the global change in vegetation growth between 1982 and 2008 [79,80]. The spatial distribution pattern of SR, WVP, and WS in East Asia is shown in Figure 5. The distribution maps of SR, WVP, and WS show that spring, autumn, and winter are very dry seasons in East Asia. Therefore, lower crop yields in the future will lead to higher PET due to increasing trends in crop water requirements [81,82].

**Figure 6:**
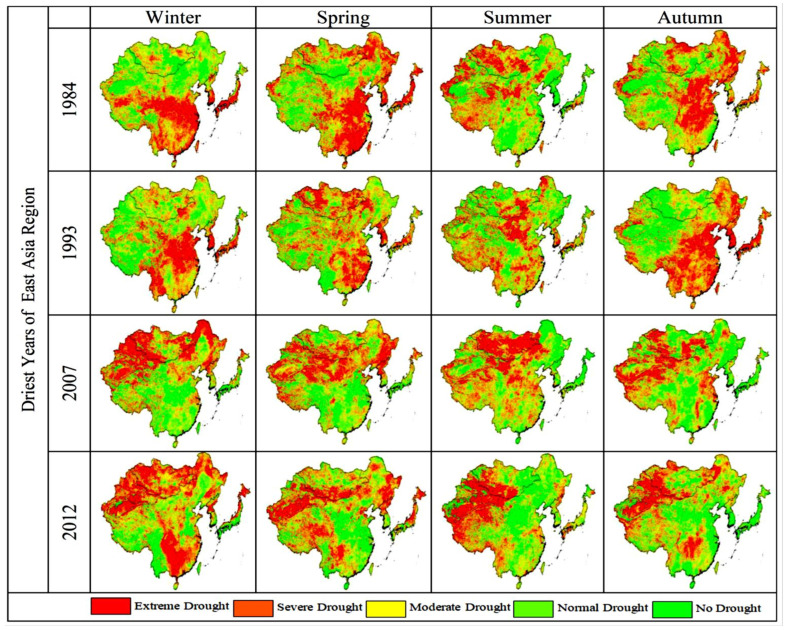
Spatial distribution of seasonal average VCI values over East Asia.

**Figure 7:**
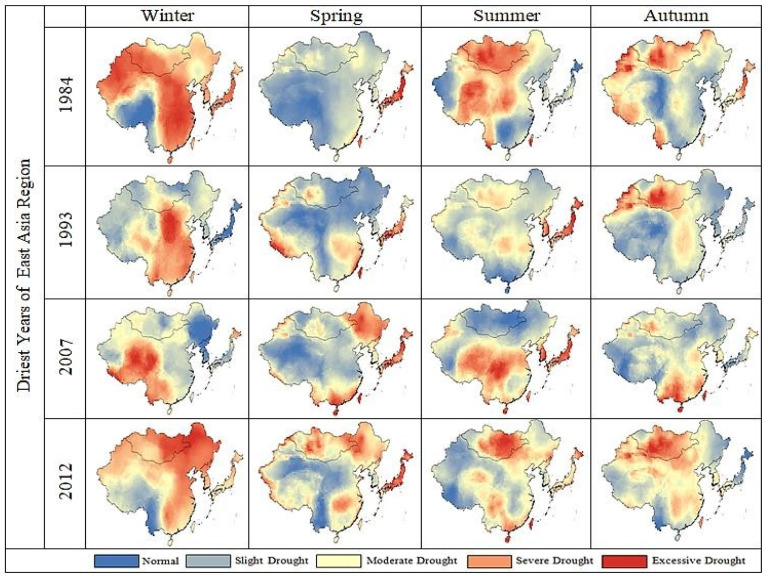
Spatial distribution of seasonal average TCI values over East Asia.

**Figure 8:**
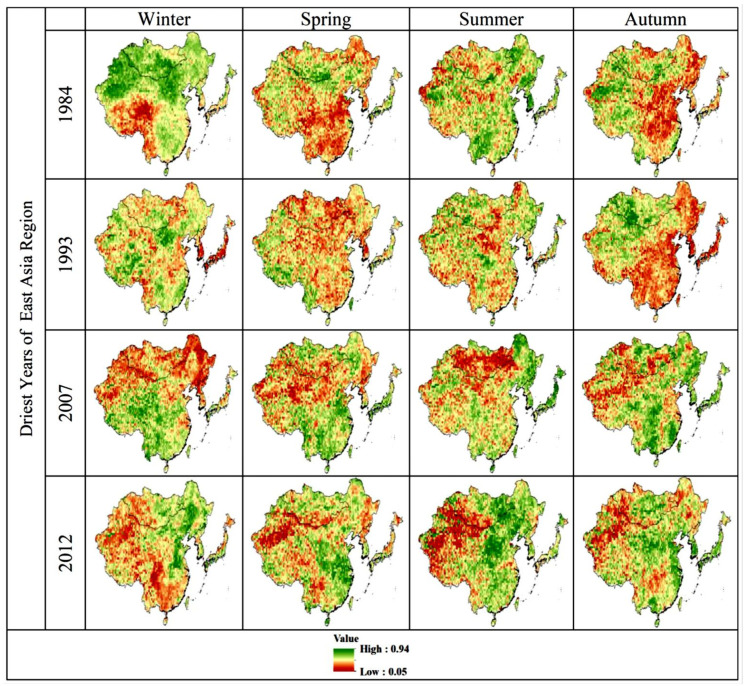
Spatial distribution of seasonal average VHI values over East Asia.

Although the NDVI values themselves do not reflect a non-dry or dry environment, measuring the deviation between the current NDVI and normal conditions can indicate the severity of the drought [83,84]. In the current study, VCI values are zero, indicating low vegetation associated with dry climates (Figure 6). These seasonally averaged VCI, TCI, and VHI values have been identified and evaluated to classify the arid regions of East Asia. Seasonal averages of VCI, TCI, and VHI values show general vegetation dynamics over the period 1982–2018. These findings are consistent with those reported by [85,86]. Water accessibility and crop yields may decrease in the future, taking advantage of increased temperatures and changes in precipitation [87,88]. Throughout the study period, average VCI, TCI, and VHI were generated seasonally, indicating the extremely arid regions of East Asia (Figure 6, Figure 7 and Figure 8). Vegetation in southern East Asia is also on the rise. However, due to the rapid urbanization process, the coherence in the southeastern region of East Asia is weak [89]. Several studies have found that VCI, TCI, and VHI respond to climatic factors such as precipitation and temperature and can be used to monitor climatic drought [90]. The spatial map shows that the East Asian Winter (DJF) is very dry. The summer (JJA) season has the highest VCI, TCI, and VHI values. In the season from spring to summer, the distribution values of VCI, TCI, and VHI show an upward trend, while in autumn, the distribution values of VCI, TCI, and VHI reach their maximum values as compared with the winter value. A gradient of surface circumstances such as temperature is caused by major changes in LST, which can play a vital role in atmospheric variations [91]. In the months that followed, low precipitation set in and dried up again, covering much of East Asia. Song et al. [68] reported that East Asia was hit by severe drought in winter, and Huang et al. [52] also reported that drought in the northwestern region was more severe than in the southeastern region. Relatively low temperatures in the spring can also adversely affect vegetation growth [92].

### 2.7. Stage Characteristics of Seasonal Temporal SPEI in Sub-Regions of East Asia

**Figure 9:**
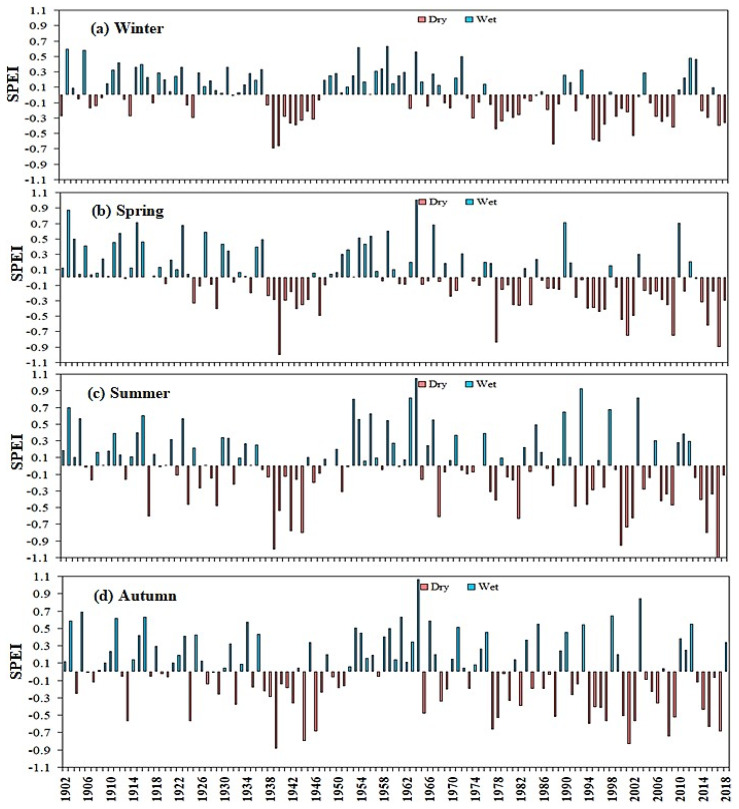
Variation of the seasonal SPEI of (**a**) winter, (**b**) spring, (**c**)summer, and (**d**) autumn, in East Asia from 1902 to 2018.

**Figure 10:**
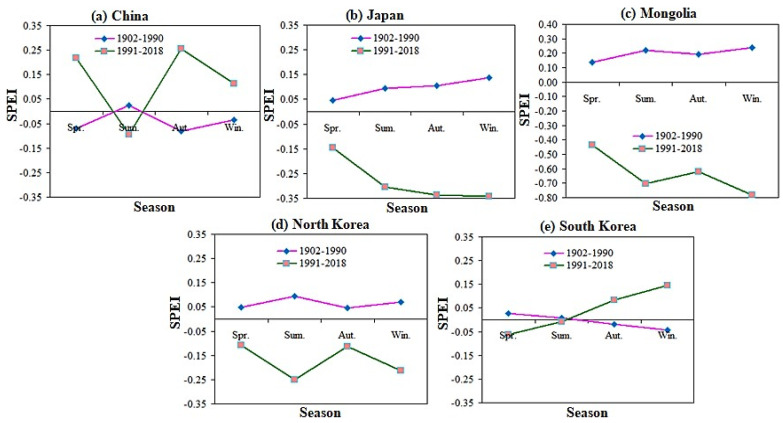
Stage characteristics of seasonal SPEI in all countries of East Asia region.

The seasonal variation of SPEI has no obvious drying trend in summer and autumn. However, East Asia showed a drought trend in winter and spring (Figure 9). Figure 10 shows the results from the division of SPEI seasons in various countries in East Asia. The mutation years are 1902–1990 and 1991–2018, respectively. Results showed that drought was most sensitive to longer SPEI timescales, suggesting that drought parameters have a greater influence on longer timescales than on shorter ones [70,71].

According to the mutation year and the stage characteristics of seasonal SPEI, the study period of each subregion in East Asia was divided. From 1902 to 1990, the SPEI in East Asian countries except South Korea indicated an increasing trend from spring to summer, and the lowest value appeared in autumn and winter when the drought was the most severe except in Mongolia and Japan. The seasonal SPEI from 1902 to 1990 showed different characteristics, with obvious wetter trends in Japan, Mongolia, and Korea and drier trends in China and Korea among the four growing seasons. During the study year 1902–1990, the SPEI values were positive in four different growing seasons in East Asia, except for China and Korea. From 1991 to 2018, the changing trends in East Asian countries were inconsistent. Except for China and South Korea, the four different growing seasons in East Asia were the most severe droughts, and the SPEI value was negative.

From 1991 to 2018, the drought in autumn and winter was the most severe in China, Japan, Mongolia, and North Korea, and the drought in autumn and winter in China and South Korea was the lightest. The trend from 1902 to 1990 is quite special. Except for China and South Korea in spring, autumn and winter, there is no drought in the four different growing seasons in East Asia, and the SPEI value is the largest positive value. However, the seasonal SPEI in 1991–2018 shows Japan, Mongolia, and North Korea. There is an obvious dry trend in different growing seasons, while China and South Korea show a wet trend in spring, autumn, and winter. Under this condition, future studies based on multi-source climate data and actual PET and SPEI data could lower the potential for inaccuracies and strengthen the conclusions. Our next step is to develop new methods with higher resolution for downscaling.

## 3. Study Region and Data Analysis

### 3.1. Study Area

The East Asia region contains Mongolia, China, North Korea, South Korea, and Japan, from 5° N to 55° N and 70° E to 140° E, with an elevation of 1103 m (3 619 feet) and a barometric pressure of 89 kPa. The study region covers an area of about 5,125,000 km^2^. This region includes a variety of climatic zones, including arid, tropical, subtropical, temperate, continental, water, and arid regions. The main vegetation in East Asia consists of crops, short grass, evergreen trees, deciduous trees, tall grass, evergreen shrubs, deciduous shrubs, and mixed trees.

### 3.2. Datasets

**Figure 11:**
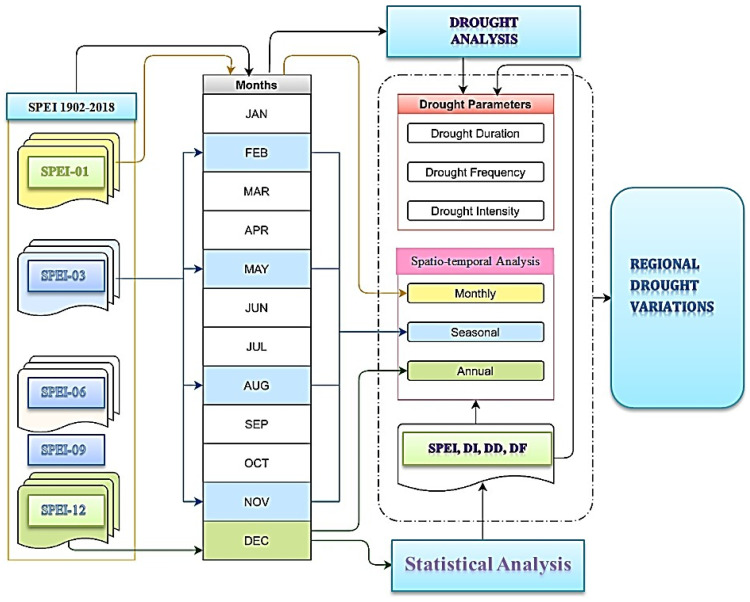
Flowchart of study.

**Figure 12:**
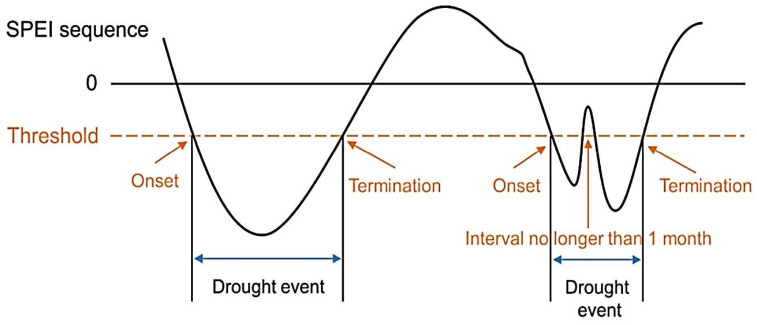
Schematic diagram of SPEI time series.

**Figure 13:**
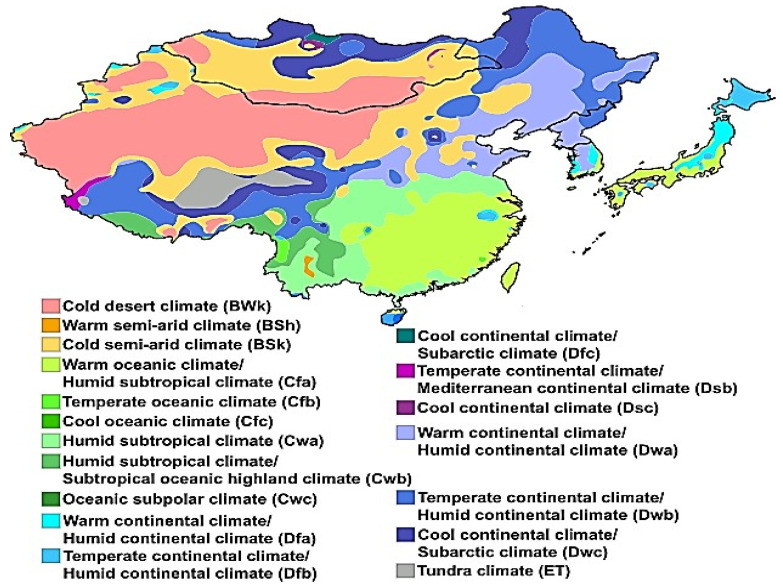
East Asia region climate classifications.

We use the multi-scale SPEI matrix dataset (https://spei.csic.es/database.html) for drought identification and assessment. SPEI has been used to monitor and assess meteorological droughts [45]. In the early 1980s, scientists used AVHRR-derived NDVI on polar-orbiting satellites of the NOAA to monitor and assess terrestrial vegetation vitality [46]. Subsequent models from the GIMMS Generation 3 NDVI (bimonthly) dataset were obtained from the AVHRR sensor at a spatial resolution of 0.08330 at 15-day intervals from 1982 to 2018. Long-term monthly mean temperature data were retrieved from the Modern Research and Applications Review Analysis (MERRA by NASA). Figure 11 and Figure 12 show flow charts for the study area and a schematic diagram for identifying drought events in the SPEI time series, while Figure 13 shows climate classifications in East Asia. We obtained monthly solar radiation (kJ m^−2^ day^−1^), water vapor pressure (kPa), and wind speed (m s^−1^) data at 30 s 1 km^2^ spatial resolution from the Worldclim 2.1 database (https://www.worldclim.org/data/worldclim21.html). All the spatial and temporal analysis and calculations were performed in GIS 10.7.

### 3.3. Standardized Precipitation Evapotranspiration Index (SPEI)

Monthly SPEI (SPEI-01) can reflect slight changes in short-term drought, seasonal SPEI (SPEI-03, SPEI-06, and SPEI-09) can reflect seasonal drought conditions, and annual SPEI (SPEI-12) can reflect drought year-to-year variation and is characterized by multiple timescales. In addition, SPEI takes temperature into account and addresses the effects of changes in surface ET on drought.

### 3.4. Drought Characterization

Drought is mainly characterized by various parameters, such as duration, frequency, and intensity. SPEI values below the threshold (−1) point out drought stress [45]. In this study, SPEI values on multiple timescales, namely 1 month (SPEI-01), 3 months (SPEI-03), 6 months (SPEI-06), 9 months (SPEI-09), and 12-month (SPEI-12), were used to study the spatiotemporal pattern of drought. SPEI-01 and SPEI-03 help recognize short-term droughts, while, SPEI-06, SPEI-09, and SPEI-12 can be used to assess long-term droughts (Table 4) [47].

### 3.5. Drought Frequency (DF)

The total number of drought events that occurred within the specified time interval was considered the DF [48].

(1) $$\mathrm{DF}=\frac{n_{m}}{N_{m}}\times 100\%$$

where n_m_ is the number of drought years, and N_m_ is the total number of years.

### 3.6. Drought Duration (DD)

DD was determined as the ratio between the total duration of the entire drought event and the number of all drought events [49].

(2) $$\mathrm{DD}=\frac{\sum_{i=1}^{n}d_{i}}{n}$$

where d_i_ = duration of the ith drought; n = total number of drought events.

### 3.7. Drought Intensity (DI)

DI is defined as the cumulative shortage with a drought index less than its threshold during a drought event [50].

(3) $$\mathrm{DI}=\left|\frac{1}{n}\sum_{i=1}^{n}{\mathrm{SPEI}}_{i}\right|$$

where n is the number of drought occurrences; SPEI_i_ = cumulative SPEI value less than the threshold.

### 3.8. Vegetation Condition Index (VCI)

The VCI recommends normalizing NDVI relative to the minimum and maximum NDVI values. The VCI was calculated with the following formula [51].

(4) $$\mathrm{VCI}=\frac{\mathrm{NDVI}-{\mathrm{NDVI}}_{\min}}{{\mathrm{NDVI}}_{\max}-{\mathrm{NDVI}}_{\min}}$$

where NDVI_min_ and NDVI_max_ are the minimum and maximum values of NDVI for each pixel, respectively.

### 3.9. Temperature Condition Index (TCI)

The TCI is determined by the following equation [52].

(5) $$\mathrm{TCI}=\frac{{\mathrm{LST}}_{\max}-{\mathrm{LST}}_{i}}{{\mathrm{LST}}_{\max}-{\mathrm{LST}}_{\min}}$$

where LST_max_ and LST_min_ are the values of the maximum LST and minimum LST of each pixel, respectively, in the same month during 1982–2018.

### 3.10. Vegetation Health Index (VHI)

The VHI was calculated by the following equation between 1982 and 2018.

(6) $$\mathrm{VHI}=0.5\;{\left(\mathrm{VCI}\right)}_{\mathrm{ijk}}+0.5\;{\left(\mathrm{TCI}\right)}_{\mathrm{ijk}}$$

where monthly VCI and TCI for each i pixel, j month, and k year. The values of VCI and TCI were classified into five categories (Table 5).

## 4. Conclusions

Under climate change and global warming, significant improvements have been made in drought frequency and duration. We compared several drought indices before using the long-term SPEI to analyze drought dynamics (frequency, duration, and intensity) in East Asia over 12 decades from 1902 to 2018. Results showed that the SPEI has significantly increased the extreme drought in East Asia; this mostly affects the SPEI-06 and SPEI-12 growing seasons during 1981–2018. The dry and wet annual SPEI trends mostly affect the five subregions of East Asia; the annual SPEI had two extremely dry spells from 1936 to 1947 and from 1978 to 2018. Japan, South Korea, and North Korea are wet in the summer compared to other regions of East Asia, with drought frequency occurring at 51.4%, respectively. The mean drought frequencies in China and Mongolia are 57.4% and 54.6%. China and Mongolia are the driest regions in East Asia due to their high drought frequency and duration. The spatial seasonal analysis of solar radiation (SR), water vapor pressure (WVP), wind speed (WS), vegetation condition index (VCI), temperature condition index (TCI), and vegetation health index (VHI) confirmed that the East Asia region suffered from maximum drought events. The winter and spring showed a dry trend in the East Asia region. During 1902–1990, a seasonal SPEI presented diverse characteristics, with clear wet trends in Japan, Mongolia, and North Korea during four growing seasons and dry tends in China and South Korea. During 1991–2018, seasonal SPEI presented clear dry trends in Japan, Mongolia, and North Korea during four growing seasons, while China and South Korea showed a wet trend during the spring, autumn, and winter seasons. Our results will help to advance our understanding of climate change, the role of long-term SPEI, and its impact on drought dynamics in East Asia.

## Figures and Tables

**Table 1:** Drought frequency (DF) of various countries of East Asia with different growing seasons at different SPEI timescales in percentage (%).

East Asia Region					Drought Frequency (%)							
	1902–1990				1991–2018				1902–2018			
	Winter	Spring	Summer	Autumn	Winter	Spring	Summer	Autumn	Winter	Spring	Summer	Autumn
China	51.8	59.0	57.7	56.3	58.6	47.6	56.5	55.4	53.4	56.3	57.4	56.1
Japan	50.7	54.7	55.1	56.4	48.2	40.2	39.3	37.8	50.1	51.2	51.4	51.9
Mongolia	59.3	62.9	64.3	64.9	30.1	22.3	23.8	17.3	52.3	53.2	54.6	53.5
North Korea	51.1	53.3	52.2	51.2	45.2	40.5	47.9	44.0	49.7	50.2	51.1	49.4
South Korea	50.8	52.0	51.1	50.4	47.9	47.0	53.6	52.4	50.1	50.8	51.7	50.9

**Table 2:** Drought duration of various countries of East Asia with different growing seasons at different SPEI timescales in months.

East Asia Region					Drought Duration (Month)							
	1902–1990				1991–2018				1902–2018			
	Winter	Spring	Summer	Autumn	Winter	Spring	Summer	Autumn	Winter	Spring	Summer	Autumn
China	2.07	2.44	2.36	2.29	2.42	1.91	2.30	2.24	2.15	2.29	2.35	2.28
Japan	2.03	2.21	2.23	2.29	1.93	1.67	1.65	1.61	2.01	2.05	2.06	2.08
Mongolia	2.46	2.70	2.80	2.85	1.43	1.29	1.31	1.21	2.10	2.14	2.20	2.15
North Korea	2.05	2.14	2.09	2.05	1.83	1.68	1.92	1.79	1.99	2.01	2.05	1.98
South Korea	2.03	2.08	2.05	2.02	1.92	1.89	2.15	2.10	2.00	2.03	2.07	2.03

**Table 3:** Drought intensity of various countries of East Asia with different growing seasons at different SPEI timescales.

East Asia Region					Drought Intensity							
	1902–1990				1991–2018				1902–2018			
	Winter	Spring	Summer	Autumn	Winter	Spring	Summer	Autumn	Winter	Spring	Summer	Autumn
China	0.48	0.41	0.42	0.44	0.41	0.52	0.43	0.45	0.47	0.44	0.43	0.44
Japan	0.49	0.45	0.45	0.44	0.52	0.60	0.61	0.62	0.50	0.49	0.49	0.48
Mongolia	0.41	0.37	0.36	0.35	0.70	0.78	0.76	0.83	0.48	0.47	0.45	0.47
North Korea	0.49	0.47	0.48	0.49	0.55	0.60	0.52	0.56	0.50	0.50	0.49	0.51
South Korea	0.49	0.48	0.49	0.50	0.52	0.53	0.46	0.48	0.50	0.49	0.48	0.49

**Table 4:** Wet and dry classification scales of Standardized Precipitation Evapo-transpiration Index (SPEI) based on the index value.

Grade	Classification	SPEI Values
1	Extremely wet	≥SPEI 2.0
2	Severely wet	1.5 > SPEI ≥ 2.0
3	Moderately wet	1.0 > SPEI ≥ 1.5
4	Slight wet	0.5 > SPEI ≥ 1.0
5	Normal	−0.5 < SPEI ≥ 0.5
6	Mild drought	−1.0 < SPEI ≤ −0.5
7	Moderate drought	−1.5 < SPEI ≤ −1.0
8	Severely dry	−2.0 < SPEI ≤ −1.5
9	Extremely dry	SPEI ≤ −2.00

**Table 5:** Classification of VCI and TCI drought index.

Drought Indices Values	Drought Conditions
<0.20	Extreme drought
0.20–0.30	Severe drought
0.30–0.50	Moderate drought
0.50–0.60	Normal drought
>0.60	No drought

## Data Availability

The data that support the findings of this study are available from the corresponding author upon reasonable request.
